# A Single Dose of LSD Does Not Alter Gene Expression of the Serotonin 2A Receptor Gene (*HTR2A*) or Early Growth Response Genes (*EGR1-3*) in Healthy Subjects

**DOI:** 10.3389/fphar.2017.00423

**Published:** 2017-06-28

**Authors:** Patrick C. Dolder, Edna Grünblatt, Felix Müller, Stefan J. Borgwardt, Matthias E. Liechti

**Affiliations:** ^1^Division of Clinical Pharmacology and Toxicology, Department of Biomedicine and Department of Clinical Research, University Hospital Basel and University of BaselBasel, Switzerland; ^2^Department of Child and Adolescent Psychiatry and Psychotherapy, Psychiatric Hospital, University of ZurichZurich, Switzerland; ^3^Neuroscience Center Zurich, University of Zurich and ETH ZurichZurich, Switzerland; ^4^Zurich Center for Integrative Human Physiology, University of ZurichZurich, Switzerland; ^5^Department of Psychiatry (Universitäre Psychiatrische Kliniken Basel), University of BaselBasel, Switzerland

**Keywords:** LSD, serotonin receptor, gene expression, healthy subjects

## Abstract

**Rationale:** Renewed interest has been seen in the use of lysergic acid diethylamide (LSD) in psychiatric research and practice. The repeated use of LSD leads to tolerance that is believed to result from serotonin (5-HT) 5-HT_2A_ receptor downregulation. In rats, daily LSD administration for 4 days decreased frontal cortex 5-HT_2A_ receptor binding. Additionally, a single dose of LSD acutely increased expression of the early growth response genes *EGR1* and *EGR2* in rat and mouse brains through 5-HT_2A_ receptor stimulation. No human data on the effects of LSD on gene expression has been reported. Therefore, we investigated the effects of single-dose LSD administration on the expression of the 5-HT_2A_ receptor gene (*HTR2A*) and *EGR1-3* genes.

**Methods:** mRNA expression levels were analyzed in whole blood as a peripheral biomarker in 15 healthy subjects before and 1.5 and 24 h after the administration of LSD (100 μg) and placebo in a randomized, double-blind, placebo-controlled, cross-over study.

**Results:** LSD did not alter the expression of the *HTR2A* or *EGR1-3* genes 1.5 and 24 h after administration compared with placebo.

**Conclusion:** No changes were observed in the gene expression of LSD’s primary target receptor gene or genes that are implicated in its downstream effects. Remaining unclear is whether chronic LSD administration alters gene expression in humans.

## Introduction

Lysergic acid diethylamide (LSD) is a well-known psychoactive substance that transiently alters mind and perception. During the last few years, renewed interest has been seen in the use of LSD in psychiatric research and practice (Liechti, 2017). Modern experimental studies in humans have reported LSD’s subjective, autonomic, and endocrine effects (Schmid et al., 2015; Carhart-Harris et al., 2016a; Dolder et al., 2016; Strajhar et al., 2016; Liechti et al., 2017), functional brain activation patterns (Carhart-Harris et al., 2016b; Lebedev et al., 2016; Tagliazucchi et al., 2016; Preller et al., 2017), and pharmacokinetics (Dolder et al., 2015b, 2017). However, many aspects of LSD’s pharmacological effects remain unclear, including the phenomenon of the rapid development of tolerance (i.e., tachyphylaxis) to its psychological and physiological effects with repeated administration (Passie et al., 2008). Early studies that employed repeated daily administration of 100 μg LSD in humans described partial tolerance with the second dose and complete tolerance with the third and subsequent doses (Cholden et al., 1955; Abramson et al., 1956; Belleville et al., 1956). The effects of LSD reappeared only after a substance-free interval of 4 days. Tolerance to LSD is believed to result from serotonin 5-HT_2A_ receptor downregulation (Nichols, 2016). LSD potently binds to the 5-HT_2A_ receptor (Wacker et al., 2017) where it acts as a partial agonist (Rickli et al., 2016). Pretreatment with the 5-HT_2A_ receptor antagonist ketanserin completely prevented all perceptual and mind-altering effects of LSD in humans (Kraehenmann et al., 2017; Preller et al., 2017), indicating that the hallucinogenic effects of LSD are primarily mediated through 5-HT_2A_ receptors. Similar to humans, tolerance to the behavioral effects of LSD is also observed in rats (Buchborn et al., 2015). Consistent with a role for 5-HT_2A_ receptors in the development of tolerance, daily LSD administration for 3 days decreased 5-HT_2A_ receptor binding in the rat frontal cortex (Buckholtz et al., 1985, 1990; Gresch et al., 2005). However, another study reported only a non-significant trend toward a reduction of frontocortical 5-HT_2A_ receptor binding in rats during the development of tolerance to LSD (Buchborn et al., 2015). Additionally, no effects of acute LSD on 5-HT_2A_ receptor mRNA expression were found in the rat prefrontal cortex, hippocampus, or midbrain (Nichols and Sanders-Bush, 2002), although the effects of repeated LSD administration were not studied. Instead, adaptations in glutamate receptors were observed (Buchborn et al., 2015). Indeed, a key mechanism of action of LSD is the activation of frontal cortex glutamate transmission secondary to 5-HT_2A_ receptor stimulation (Gonzalez-Maeso et al., 2008; Moreno et al., 2011; Buchborn et al., 2015). Thus, adaptive changes that underlie tolerance to LSD may be reflected by alterations in the expression of genes that are involved in glutamatergic signaling or genes that regulate 5-HT_2A_ receptor function or its downstream signaling pathways. Acute LSD increased the expression of immediate early genes in the rat prefrontal cortex, including *EGR2* (i.e., a gene that is involved in cognition and neural plasticity) and several others genes (Nichols and Sanders-Bush, 2002; Nichols et al., 2003; Nichols and Sanders-Bush, 2004). Further animal studies showed that LSD also increased the expression of *EGR2* and *EGR1* in the mouse cortex via 5-HT_2A_ receptor stimulation (Gonzalez-Maeso et al., 2003, 2007). Finally, LSD was shown to produce a characteristic transcriptome signaling pattern in normal but not *HTR2A*^-/-^ mice (Gonzalez-Maeso et al., 2007).

Despite the renewed interest in the clinical use of LSD, no human studies on the effects of LSD on gene expression have been performed. Therefore, we investigated the effects of a single dose of LSD on the expression of the 5-HT_2A_ receptor gene (*HTR2A*) and *EGR1-3* genes. The *EGR1* and *EGR2* genes were studied based on preclinical data (Gonzalez-Maeso et al., 2003, 2007), and the *EGR3* gene was studied because it has been shown to regulate *HTR2A* expression (Maple et al., 2015).

Acute changes in gene expression cannot be determined in heathy human brain tissue, as biopsy or postmortem tissue is not available. However, mRNA level changes in blood for candidate genes including *EGR* and *HTR2A* can cautiously be used as peripheral markers of transcription alterations in the CNS in response to interventions or to characterize patient groups (Sullivan et al., 2006; Mohr and Liew, 2007; Desjardins et al., 2008; Belzeaux et al., 2010; Rollins et al., 2010; Rivera-Baltanas et al., 2014; Cattane et al., 2015). The *EGR* and *HTR2A* genes are expressed in peripheral blood cells (Stefulj et al., 2000; Fukuda et al., 2006; Inoue et al., 2011; Cattane et al., 2015). The peripheral mRNA expression of several genes including the *HTR2A* gene were shown to have relatively similar expression profiles as in brain tissues (Glatt et al., 2005; Fukuda et al., 2006; Sullivan et al., 2006; Desjardins et al., 2008; Rollins et al., 2010; Yubero-Lahoz et al., 2015; GTExPortal, 2017). In healthy humans, *HTR2A* gene expression levels in whole blood correlated with 5-HT metabolite levels in the cerebrospinal fluid (Luykx et al., 2016). Peripheral *EGR1* expression was increased in patients with schizophrenia compared with healthy controls (Cattane et al., 2015).

Therefore, we expected altered *HTR2A* expression in response to LSD. Additionally, we hypothesized that LSD acutely increases *EGR1* and *EGR2* gene expression in humans similarly to rodents.

## Materials and Methods

### Study Design

The study used a double-blind, placebo-controlled, cross-over design with two experimental test sessions in balanced order. The washout periods between sessions were at least 7 days. The study was conducted in accordance with the Declaration of Helsinki and International Conference on Harmonization Guidelines in Good Clinical Practice (ICH-GCP) and approved by the Ethics Committee of Northwestern Switzerland. The administration of LSD in healthy subjects was authorized by the Swiss Federal Office for Public Health, Bern, Switzerland. All of the subjects provided written informed consent and were paid for their participation. The study was registered at ClinicalTrials.gov (NCT02308969).

### Participants

Twenty-four healthy subjects (12 men, 12 women; mean age ± SD: 33 ± 11 years; range: 25–60 years) participated in the study. Blood samples for gene expression measurements were taken from only 15 participants (7 men, 8 women; mean age ± SD: 28.5 ± 5.8 years; range: 25–48 years; mean weight ± SD: 68 ± 8 kg; range: 55–85 kg; mean BMI ± SD: 22.0 ± 2.0 kg/m^2^; range: 19–24 kg/m^2^). The inclusion and exclusion criteria, subjective, autonomic, and adverse effects of LSD, and pharmacokinetic data from this study have been reported in detail elsewhere (Dolder et al., 2016, 2017; Liechti et al., 2017). Briefly, the participants had to be 25- to 65-years old and physically and mentally healthy. Additional exclusion criteria were pregnancy, tobacco smoking (>10 cigarettes/day), life-time prevalence of illicit drug use >10 times (except tetrahydrocannabinol), and illicit drug use within the past 2 months or during the study (determined by urine drug tests). Of the 15 subjects, only two had used a hallucinogen (LSD and psilocybin) once in their lives.

### Study Procedures

The experimental sessions were conducted in a standard hospital patient room. The participants were resting in hospital beds except when going to the restroom. Only one research participant and one or two investigators were present during the experimental sessions. The participants could interact with the investigator, rest quietly, and/or listen to music via headphones, but no other entertainment was provided. LSD or placebo was administered at 9:00 AM. A standardized lunch and dinner were served at 1:30 and 5.30 PM, respectively. The subjects were never alone during the first 12 h after drug administration, and the investigator was in a room next to the subject for up to 24 h while the subject was asleep (mostly from 1:00 to 8:00 AM).

### Study Drug

LSD (D-LSD, Lipomed AG, Arlesheim, Switzerland) was administered in a single oral dose of 100 μg as a capsule. The dose was within the range of doses that are taken for recreational purposes (Passie et al., 2008; Nichols, 2016). Corresponding placebo capsules were used.

### Measures

#### Gene Expression

Blood samples were collected before and 1.5 and 24 h after drug administration using the PAXgene^TM^ Blood RNA system (Becton Dickinson, Heidelberg, Germany). The 1.5 h time point was selected to coincide with the peak of the plasma concentration of LSD (Dolder et al., 2015b). The 24 h time point was selected because partial tolerance by that time has been documented (Cholden et al., 1955; Abramson et al., 1956; Belleville et al., 1956). Samples were incubated for 2 h at room temperature, followed by freezing at -80°C until further processing. Total RNA was prepared using the PAXgene^TM^ Blood RNA Kit 50 (PreAnalytiX, Qiagen, Hilden, Germany). Total RNA samples were spectrophotometrically scanned (260 and 280 nm; NanoVue, GE Healthcare Life Sciences, Glattbrugg, Switzerland). A260 was used for RNA quantification. The A260/A280 ratio was >1.9, excluding relevant protein contamination. RNA quality was also measured using Experion RNA chips (BioRad, Hercules, CA, United States) providing the RNA quality indicator (RQI > 7). Quantitative real-time polymerase chain reaction (PCR) was performed for the *HTR2A*, *EGR1*, *EGR2*, and *EGR3* genes and six additional reference genes (*ACTB*, *GAPDH*, *ALAS1*, *RPL13A*, *PPIA*, and *RRN18S*) as described previously (Grunblatt et al., 2009). The investigated genes are listed in detail in **Table 1**. Total RNA (500 ng) from each sample was reverse-transcribed using the iScript cDNA synthesis kit (BioRad, Hercules, CA, United States). Each amplification was performed in a total volume of 10 μl that contained 5 μl of the QuantiFast SYBR Green PCR kit (Qiagen, Hilden, Germany) and the specific PrimerAssay (Qiagen, Hilden, Germany). The PCR conditions were run on a CFX384 device (BioRad, Hercules, CA, United States) according to manufacturer’s manual, with the exception of *HTR2A* primers, in which annealing occurred at 56°C according to a gradient analysis (Qiagen, Hilden, Germany). A melting-point analysis was conducted for each assay to confirm the specificity of the PCR products. All of the PCR reactions were run in triplicate. LinRegPCR 2016.0 software (Hart Failure Research Center, Amsterdam, The Netherlands; Ramakers et al., 2003) was used to determine the PCR efficiency. The analysis of gene expression and normalization with the most stable reference genes was conducted using qBasePlus 3.0 software (Biogazelle, Gent, The Netherlands; Vandesompele et al., 2002). Since the consensus is that there are no real reference (housekeeping) genes, there is a need to use more than one reference genes as well as test them before normalization is conducted (Vandesompele et al., 2002). The reference genes *GAPDH* and *PPIA* were the least stable and thus excluded, and the normalization analysis was conducted using the four remaining reference genes (*ACTB*, *ALAS1*, *RPL13A*, and *RRN18S*).

**Table 1:** List of investigated genes.

Gene name	Abbreviation	Qiagen (cat. no.)	Gene bank accession no.
**Reference genes**			
β-Actin	ACTB^∗^	QT00095431	NM_001101
Glyceraldehydes-3-phosphate dehydrogenase	GAPDH	QT00079247	NM_002046
Aminolevulinate delta synthase 1	ALAS1^∗^	QT00073122	NM_000688
Ribosomal protein L13a	RPL13A^∗^	QT00089915	NM_012423
Peptidylprolyl isomerase A	PPIA	QT00052311	NM_021130
18s ribosomal	RRN18S^∗^	QT00199367	V01270
**Genes of interest**			
Serotonin 2a receptor	HTR2A	QT00054306	NM_000621
Early growth response 1	EGR1	QT00218505	NM_001964
Early growth response 2	EGR2	QT00000924	NM_000399
Early growth response 3	EGR3	QT00246498	NM_001199880
			NM_001199881 NM_004430


*^∗^Most stable reference genes used for normalization.*

#### Plasma Concentrations of LSD

Blood was collected in lithium heparin tubes before and 1, 2, 3, 4, 6, 8, 10, 12, 16, and 24 h after LSD administration. The blood samples were immediately centrifuged, and the plasma was rapidly stored at -20°C and later at -80°C until analysis. LSD concentrations in plasma were determined using liquid-chromatography-tandem mass-spectrometry as reported in detail elsewhere (Dolder et al., 2015a; Steuer et al., 2016). The lower limit of quantification was 0.05 ng/ml (Dolder et al., 2015a). The pharmacokinetics of LSD from the present study are presented in detail elsewhere (Dolder et al., 2017).

### Statistics

The statistical analyses were performed using Statistica 12 software (StatSoft, Tulsa, OK, United States). Baseline gene expression values before drug administration were set to 1, and changes after 1.5 and 24 h are expressed as fold changes from baseline. Differences between LSD and placebo at the corresponding time points were then analyzed using paired *t*-tests. All comparisons were also made with data standardized to the mean age, body weight, and peak plasma concentrations of LSD. The criterion for significance was *p* < 0.05 without correction for multiple comparisons. Additionally, to test for changes in gene expression over time after administration of LSD or placebo, repeated measures analyses of variance (ANOVAs) were conducted with time (0, 1.5, and 24 h) as within-subject factor followed by Tukey *post hoc* test. To assess potential moderating effects by sex, sex was added as additional between-subjects factor to the ANOVAs.

## Results

**FIGURE 1:**
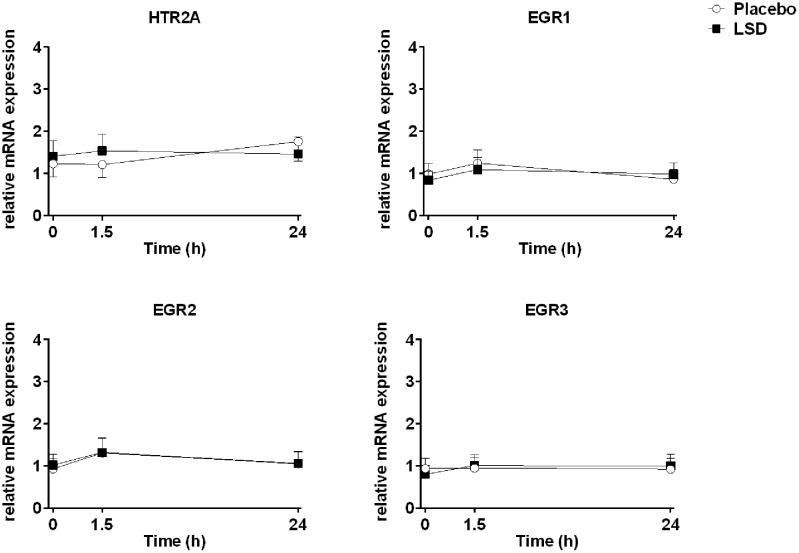
Lysergic acid diethylamide did not alter gene expression. The levels of expression of the 5-HT_2A_ receptor gene (*HTR2A*) and early growth response genes (*EGR1*, *EGR2*, and *EGR3*) were determined before and 1.5 and 24 h after administration of 100 μg LSD or placebo. The data are expressed as the mean ± SEM of mRNA expression levels relative to reference genes with stable expression. The respective differences in fold-changes from baseline are show in **Table 2**.

Three samples after 24 h had insufficient amounts of RNA to be included in the analysis (**Table 2**). The expression patterns of the *HTR2A*, *EGR1*, *EGR2*, and *EGR3* genes were unchanged 1.5 and 24 h after the administration of LSD compared with placebo (**Figure 1** and **Table 2**). Expression did also not change over time after LSD and placebo (HTR2A: *F*_2,24_ = 0.02, *P* = 1.0 and *F*_2,26_ = 1.24, *P* = 0.3; EGR1: *F*_2,24_ = 1.14, *P* = 0.3 and *F*_2,26_ = 1.9, *P* = 0.2; EGR2: *F*_2,24_ = 1.20, *P* = 0.3 and *F*_2,26_ = 2.67, *P* = 0.09; EGR3: *F*_2,24_ = 1.17, *P* = 0.2 and *F*_2,24_ = 0.08, *P* = 0.9, respectively). Sex did not moderate the effects of LSD or placebo on gene expression. Additionally, the findings were similar if the data was standardized to the mean age, body weight or plasma concentration of LSD.

**Table 2:** Lysergic acid diethylamide-induced changes in gene expression.

Gene	Time after LSD	*n*	LSD vs. placebo^a^	*p*-value^b^
*HTR2A*	1.5 h	15	0.51 (0.13)	0.39
	24 h	12	0.07 (0.02)	0.90
*EGR1*	1.5 h	15	-0.1 (-0.03)	0.83
	24 h	12	0.47 (0.14)	0.10
*EGR2*	1.5 h	15	0.04 (0.01)	0.86
	24 h	12	0.27 (0.08)	0.22
*EGR3*	1.5 h	15	0.20 (0.05)	0.24
	24 h	11	0.40 (0.12)	0.16


**HTR2A*, serotonin 2a receptor gene; *EGR*, early growth response gene; n, number of samples. ^a^Gene expression values are differences (mean and [SEM]) between LSD and placebo at the respective time point for fold-changes from baseline; ^b^*t*-tests.*

## Discussion

The key finding of the present study was that acute LSD administration did not alter the expression of the *HTR2A* and *EGR1-3* genes in humans using peripheral blood cells as peripheral biomarker possibly reflecting central gene expression. The lack of an acute effect of LSD on *HTR2A* gene expression in humans is consistent with a study in rats that reported no changes in *HTR2A* gene expression in different brain areas (Nichols and Sanders-Bush, 2002). However, 5-HT_2A_ receptor availability may also be altered independently of *HTR2A* gene expression (e.g., by receptor internalization or moderation of its activity). Several studies in rats (Buckholtz et al., 1985, 1990; Gresch et al., 2005; Buchborn et al., 2015) reported a decrease in 5-HT_2A_ receptor binding in the prefrontal cortex or consistent trend effects (Buchborn et al., 2015) after repeated LSD administration. Unknown, however, is whether lower binding also occurs after single-dose administration. The present findings of no changes in *EGR1* and *EGR2* gene expression in human blood samples after acute LSD administration contrast with preclinical findings. Specifically, LSD rapidly increased *EGR1* and *EGR2* expression in the cortex in rats (Nichols and Sanders-Bush, 2002, 2004; Nichols et al., 2003) and mice (Gonzalez-Maeso et al., 2003, 2007). We expected similar rapid increases in *EGR1* and *EGR2* expression in humans. Importantly, however, we evaluated gene expression in human blood samples, whereas the animal studies evaluated gene expression in brain tissue. Thus, it is possible that LSD alters gene expression in the brain and not in blood.

Tolerance to repeated LSD administration reportedly begins with the second daily dose of LSD, and complete tolerance develops within 3–4 days of repeated LSD administration in humans according to older studies (Cholden et al., 1955; Abramson et al., 1956; Belleville et al., 1956; Passie et al., 2008; Nichols, 2016) that need to be replicated. In the present study, we found no evidence of acute pharmacological tolerance within 12 h of acute LSD administration at a dose of 100 μg as documented in detail elsewhere (Dolder et al., 2017). Similarly, no acute tolerance was observed after single-dose administration of 200 μg LSD in humans within 24 h (Dolder et al., 2015b, 2017). Thus, after one dose of LSD, subjective effects of LSD were self-reported by the participants as long as LSD was present in plasma, and the subjective effects did not decline more rapidly than the plasma concentrations of LSD (Dolder et al., 2017). This is consistent with the view that LSD directly activates 5-HT_2A_ receptors to produce its mind-altering effects as long as it is present in the effect compartment (i.e., the brain) and assuming largely similar plasma and effect compartment kinetics. The finding of no acute tolerance in the participants in the present study (Dolder et al., 2017) also indicates that no relevant counterregulatory neuroadaptations occurred or were evident with the first 12–24 h after LSD administration. A recent study showed that LSD dissociates very slowly from the 5-HT_2A_ receptor, and the authors proposed that the high potency and long effect duration of LSD could be linked to a unique receptor interaction (Wacker et al., 2017). However, the LSD concentration-effect relationship (Dolder et al., 2017) shows that the presence of LSD in the body sufficiently accounted for the duration of its subjective effects. Doubling the LSD dose resulted in prolongation of the effect by approximately 3 h, consistent with its half-life of approximately 3 h (Dolder et al., 2017). In contrast to the pharmacokinetic–pharmacodynamic relationship of LSD (Dolder et al., 2017), other psychoactive substances, such as 3,4-methylenedioxymethamphetamine (MDMA), exhibit very marked acute pharmacological tolerance, with a rapid decline of subjective and physiological effects of MDMA within 4 h despite continuously high plasma levels and a relatively long half-life of 8 h (Hysek et al., 2010; Vizeli and Liechti, 2017).

## Limitations

The present study has several limitations. First, we assessed the effects of only an acute single dose of LSD on gene expression and tolerance. Further repeated dose administration studies need to be conducted. Second, we used only a moderate single dose of LSD, and the study sample was relatively small. Third, we assessed gene expression only at 1.5 and 24 h after LSD administration. Therefore, we may have missed effects that may have occurred between these time points. In fact, increases in *EGR2* were observed only up to 5 h in rats (Nichols and Sanders-Bush, 2002; Gonzalez-Maeso et al., 2003; Nichols et al., 2003). Fourth, all changes in gene expression that are caused by LSD that have been reported to date have been observed animal brains, whereas our study focused solely on human blood cells as a peripheral biomarker of the central nervous system (Sullivan et al., 2006; Mohr and Liew, 2007; Desjardins et al., 2008; Belzeaux et al., 2010; Rollins et al., 2010; Rivera-Baltanas et al., 2014; Cattane et al., 2015).

## Conclusion

In summary, an acute single dose of LSD in humans did not acutely alter the expression of the *HTR2A* and *EGR1-3* genes in peripheral mononuclear blood cells and thus did not influence potential markers of neuroadaptation.

## Author Contributions

Each of the authors participated in this research by contributing to the conception and design of the study (PD and ML), study management (PD, FM, and SB) performance of laboratory experiments (EG) and statistical analysis and interpretation (PD, EG, and ML).

## Conflict of Interest Statement

The authors declare that the research was conducted in the absence of any commercial or financial relationships that could be construed as a potential conflict of interest.
